# Reviving Health Osekkai in Rural Japan: Collaborative Strategies of Family Physicians and Medical Students Against Social Isolation

**DOI:** 10.7759/cureus.49195

**Published:** 2023-11-21

**Authors:** Riko Herai, Ryuichi Ohta, Chiaki Sano

**Affiliations:** 1 Family Medicine, Shimane University Faculty of Medicine, Izumo, JPN; 2 Communiy Care, Unnan City Hospital, Unnan, JPN; 3 Community Medicine Management, Shimane University Faculty of Medicine, Izumo, JPN

**Keywords:** community welfare, social alienation, societal challenges, social cohesion, medical students, family physicians, rural japanese communities, community health, covid-19, osekkai

## Abstract

In the face of societal challenges exacerbated by the coronavirus disease 2019 (COVID-19) pandemic, rural Japanese communities are redoubling their efforts to preserve social cohesion. This revitalization is epitomized by the Osekkai initiative, an embodiment of voluntary neighborly support. Here, we delve into the combined efforts of family physicians and medical students in reshaping the Osekkai landscape, emphasizing the role of healthcare professionals in community health and combatting rural isolation.

## Editorial

Introduction

Japan's rural communities have historically been known for mutual assistance and community welfare [1,2]. These communities, characterized by their strong interdependence, have faced significant challenges due to demographic shifts, including aging populations and urban migration, as well as the unprecedented impact of the coronavirus disease 2019 (COVID-19) pandemic [2]. This paper explores the revival of traditional Japanese values through the Osekkai initiative, a concept rooted in voluntary mutual assistance, in these changing times.

The essence of Osekkai

Osekkai, traditionally a platform for neighborly support, extends beyond material aid to include emotional solidarity and active community participation [2,3]. This concept has historical roots in Japanese culture, where community cohesion was paramount [3]. During the COVID-19 pandemic, when isolation became a significant issue, the principles of Osekkai have gained renewed importance, facilitating societal connections and support networks in rural areas where healthcare resources are often limited [3].

Family physicians: guardians of community health

Family physicians are crucial in bridging traditional community values with modern medical practices [3]. With a comprehensive understanding of community health dynamics, they extend their services beyond clinical responsibilities to include community outreach and health education. Their involvement in the Osekkai initiative reinforces their position as pivotal figures in sustaining and improving community health, especially during challenging times like the pandemic.

Medical students: fresh perspectives and energetic involvement

Medical students contribute fresh perspectives and vitality to the Osekkai framework. Their active participation, exemplified at community gatherings such as Eve in Unnan City, represents a blend of academic learning and community service [3]. These students gain invaluable experiential knowledge while contributing to community welfare by engaging with local populations. Their collaboration with family physicians ensures the preservation of Osekkai's traditional essence while adapting it to contemporary societal needs [3,4].

The synergistic approach

The collaboration of family physicians and medical students in the Osekkai framework offers a multi-faceted approach to community health [3,4]. While family physicians provide structured, health-based perspectives, medical students introduce innovative solutions and energetic participation [3,4]. This synergy has revitalized the Osekkai concept, making it more relevant in addressing the unique challenges posed by the pandemic.

Broadening horizons

Expanding the reach of Osekkai is crucial for its continued relevance and growth. Bringing together medical students from diverse backgrounds and experienced family physicians fosters a more inclusive approach [4]. Collaborations with public entities and educational institutions highlight the importance of a comprehensive community healthcare system where traditional practices meet modern healthcare needs [4].

Addressing loneliness and social alienation

The therapeutic potential of Osekkai, particularly in combating loneliness and social alienation, has been highlighted during the pandemic. Preliminary studies indicate reduced loneliness among participants engaged in Osekkai activities [4,5]. The holistic approach taken by family physicians, coupled with the proactive involvement of medical students, positions Osekkai as an effective strategy against social isolation [2,3].

Conclusion

In an era of unprecedented societal challenges, initiatives like Osekkai stand as beacons of hope and resilience. The synergistic partnership between experienced family physicians and enthusiastic medical students presents a formidable strategy for revitalizing community health. As we navigate the aftermath of the pandemic, the lessons learned, and the collaborative efforts in these community-driven initiatives will undoubtedly shape future community healthcare strategies, emphasizing integration, innovation, and a deep-rooted concern for community welfare.

## References

[1] Ohta R, Katsube T, Sano C (2022). Challenges in help-seeking behaviors among rural older people mitigated through family physician-driven outreach: a systematic review. Int J Environ Res Public Health.

[2] Ohta R, Maiguma K, Yata A, Sano C (2022). Rebuilding social capital through Osekkai conferences in rural communities: a social network analysis. Int J Environ Res Public Health.

[3] Ohta R, Sano C (2023). Rural health dialogue for the sustainability of help-seeking behaviors among older patients: grounded theory approach. BMC Geriatr.

[4] Ohta R, Yata A, Sano C (2022). Students’ learning on sustainable development goals through interactive lectures and fieldwork in rural communities: grounded theory approach. Sustainability.

[5] Ohta R, Yata A, Sano C (2023). Healthcare profession students' motivations for learning about community organizing: a thematic analysis. Cureus.

